# Supplementary material for: Causal Relationship between Aging and Anorexia Nervosa: A White-Matter-Microstructure-Mediated Mendelian Randomization Analysis
Source: Biomedicines. 2024 Aug 16;12(8):1874. doi: 10.3390/biomedicines12081874 (PMC11351342; doi:10.3390/biomedicines12081874)
Supplement: Supplementary file 1 [file biomedicines-12-01874-s001.zip › Supplementary Figure/Figure S4/Singe SNP plot of MR analysis for TL to ubm-b-1456.pdf]

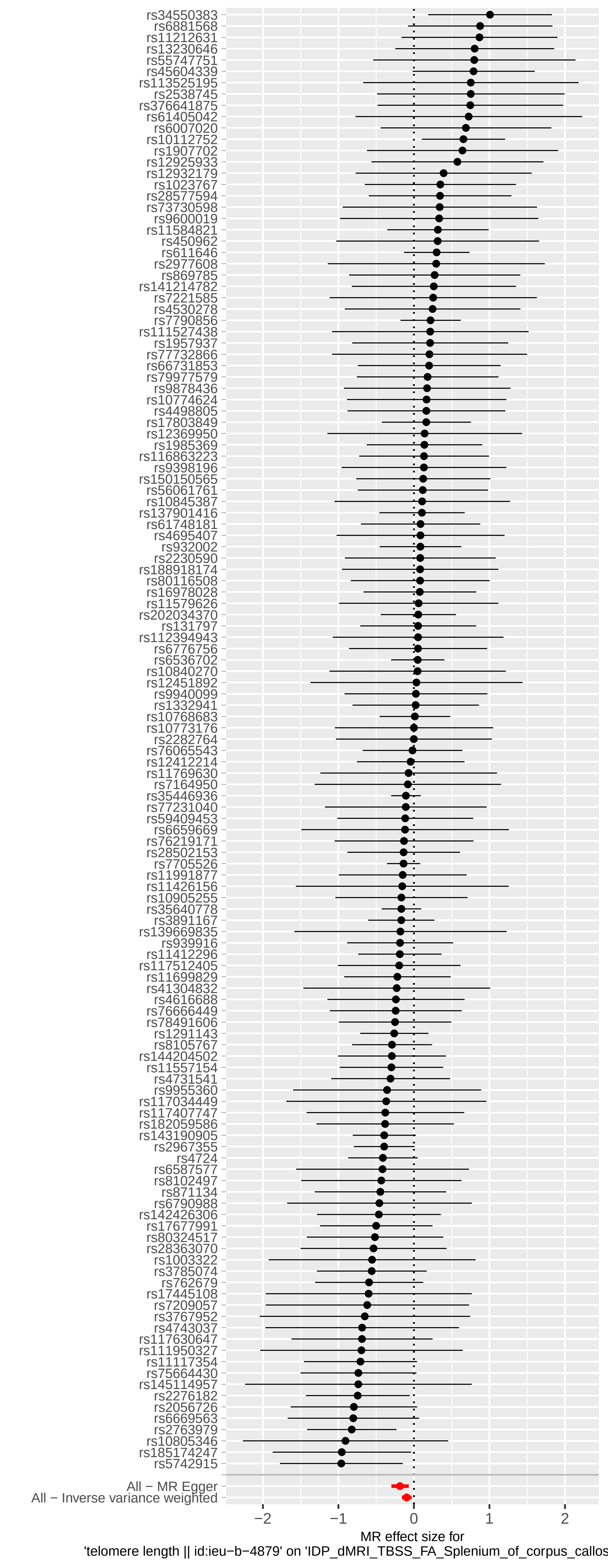
rs34550383
rs6881568
rs11212631
rs13230646
rs55747751
rs45604339
rs113525195
rs2538745
rs376641875
rs61405042
rs6007020
rs10112752
rs1907702
rs12925933
rs12932179
rs1023767
rs28577594
rs73730598
rs9600019
rs11584821
rs450962
rs611646
rs2977608
rs869785
rs141214782
rs7221585
rs4530278
rs7790856
rs111527438
rs1957937
rs77732866
rs66731853
rs79977579
rs9878436
rs10774624
rs4498805
rs17803849
rs12369950
rs1985369
rs116863223
rs9398196
rs150150565
rs56061761
rs10845387
rs137901416
rs61748181
rs4695407
rs932002
rs2230590
rs188918174
rs80116508
rs16978028
rs11579626
rs202034370
rs131797
rs112394943
rs6776756
rs6536702
rs10840270
rs12451892
rs9940099
rs1332941
rs10768683
rs10773176
rs2282764
rs76065543
rs12412214
rs11769630
rs7164950
rs35446936
rs77231040
rs59409453
rs6659669
rs76219171
rs28502153
rs7705526
rs11991877
rs11426156
rs10905255
rs35640778
rs3891167
rs139669835
rs939916
rs11412296
rs117512405
rs11699829
rs41304832
rs4616688
rs76666449
rs78491606
rs1291143
rs8105767
rs144204502
rs11557154
rs4731541
rs9955360
rs117034449
rs117407747
rs182059586
rs143190905
rs2967355
rs4724
rs6587577
rs8102497
rs871134
rs6790988
rs142426306
rs17677991
rs80324517
rs28363070
rs1003322
rs3785074
rs762679
rs17445108
rs7209057
rs3767952
rs4743037
rs117630647
rs111950327
rs11117354
rs75664430
rs145114957
rs2276182
rs2056726
rs6669563
rs2763979
rs10805346
rs185174247
rs5742915
All − MR Egger
All − Inverse variance weighted
−2
−1
0
1
2
MR effect size for
'telomere length || id:ieu−b−4879' on 'IDP_dMRI_TBSS_FA_Splenium_of_corpus_callos
